# Cultivating mathematical mindset via online video interventions: a mixed-methods investigation in Chinese higher education

**DOI:** 10.3389/fpsyg.2024.1494702

**Published:** 2025-01-07

**Authors:** Xiaoyu Xu, Jaclyn Broadbent, Qiaoping Zhang

**Affiliations:** ^1^School of Mathematics, Guangdong University of Education, Guangzhou, Guangdong, China; ^2^Centre for Research in Assessment and Digital Learning, Deakin University, Burwood, VIC, Australia; ^3^School of Psychology, Deakin University, Burwood, VIC, Australia; ^4^Department of Mathematics and Information Technology, The Education University of Hong Kong, Hong Kong, Hong Kong SAR, China

**Keywords:** mathematical mindset, calculus learning, self-regulated learning, freshmen learning, online learning

## Abstract

Freshmen often encounter significant challenges in adapting to the complexity of university-level mathematics and independent learning. These challenges necessitate the development of strong self-regulated learning (SRL) skills to successfully navigate the demands of higher education. Building on mindset theory, this study explores how mathematical mindset-oriented interventions can support freshmen’s mathematics learning during their transition to higher education mathematics, particularly in an online setting. This mixed-methods study involved 306 freshmen, who participated in an online calculus tutorial program, with 118 engaged in the mindset intervention and 188 serving as controls. The intervention significantly altered the students’ perceptions of mathematics and improved their SRL strategies. Quantitative data were analyzed using descriptive statistics, ANOVA, *t*-tests, and Structural Equation Modeling to examine the relationships between mathematical mindset, SRL, and academic achievement. Qualitative data from semi-structured interviews with 18 students were thematically analyzed to provide deeper insights into students’ experiences and mindset development. Students with a mathematical mindset demonstrated enhanced SRL strategies and superior mathematical achievement. However, the fact that some students with a fixed mindset also achieved high levels of mathematical success points to the intervention’s complex influence on academic confidence and achievement. These findings highlight the need for ongoing research into the mathematical growth mindset at the tertiary level and for adapting educational strategies to the changing dynamics of online education and diverse cultural backgrounds.

## Introduction

In higher education, freshmen often face the challenge of transitioning from high school to university learning methods, encountering numerous academic difficulties such as issues with learning effort, intrinsic motivation, self-regulated learning, and academic achievement (Freeman et al., 2007). This is particularly evident in the field of mathematics, where many freshmen struggle with low motivation and poor achievement (Tang et al., 2023). In Chinese higher education, traditional teaching methods have largely emphasized rote learning, whereas there is an increasing emphasis on fostering autonomous and self-regulated learning approaches (Li et al., 2018). This shift is further complicated by the need for students to develop higher-order thinking skills and a deeper understanding of mathematical concepts.

Calculus, a required course in many university programs, exemplifies these challenges due to its abstract and complex nature, especially for freshmen, who face increased complexity and higher expectations for self-regulated learning during their transition to university-level mathematics (Champion and Mesa, 2018; Stewart and Thomas, 2009). These difficulties can undermine students’ confidence, often leading them to develop a fixed mindset—the belief that their mathematical abilities are innate and unchangeable (Dweck et al., 1995). This mindset discourages students from investing further effort when faced with challenges, which not only impacts their immediate performance but also diminishes their long-term engagement and success in mathematics (Dweck, 2006).

While mindset interventions have shown promise in promoting student engagement, effort, and persistence, recent research suggests that these interventions alone may not be sufficient to fully address the challenges faced by freshmen, particularly in complex subjects like calculus. This indicates that mindset interventions, while effective in shifting students’ attitudes, must be complemented with other forms of support that address the practical aspects of learning complex subjects. Specifically, Boaler et al. (2021) explored a mathematical mindset summer camp and found that, although student engagement and confidence improved, the complexity of the mathematical challenges still required additional support beyond mindset change. Complementary interventions, such as self-regulated learning strategies—including goal setting, effective time management, and reflective practices—can provide the tools students need to manage their own learning and tackle complex mathematical problems, thereby offering a more comprehensive approach to improving student outcomes in mathematics (Blackwell et al., 2007; Dweck and Master, 2009; Yeager et al., 2019).

Moreover, the transition to university presents unique challenges for freshmen, as they must adapt to more complex content, increased autonomy, and higher expectations. Despite the potential benefits of mindset interventions, there is limited research specifically focusing on university freshmen, particularly in understanding how fostering a growth mindset can support their adaptation to university-level learning and mathematics achievement. This gap highlights the importance of exploring tailored interventions that address not only students’ beliefs about their abilities but also equip them with the necessary self-regulated learning strategies to succeed in their academic pursuits.

Additionally, despite the growing body of research on mathematical mindset and self-regulated learning (SRL), there remains a significant gap in understanding how these interventions function in the context of online education, particularly at the university level (Ayu, 2020; Dumford and Miller, 2018). Most studies to date have focused on traditional classroom environments, leaving the effects of online formats for delivering mindset interventions on SRL and mathematical mindset relatively unexplored (Lajoie, 2008; Cassidy, 2011; Theobald, 2021). With the increasing reliance on online learning platforms, especially during the COVID-19 pandemic, it is crucial to understand how delivering mindset interventions via online platforms can support students’ development in mathematics. The online format here is not intended as a separate intervention but as a means of delivering the mathematical mindset intervention. Online video interventions have emerged as a promising tool to address these challenges, offering flexible and interactive learning experiences (Liu et al., 2021). These interventions can provide personalized learning paths and present mathematical concepts engagingly, promoting active learning and reflection. However, despite their potential, the effectiveness of these interventions in cultivating a mathematical mindset and enhancing SRL within the context of Chinese higher education remains underexplored.

This study aims to investigate the impact of these interventions on freshmen’s SRL and mathematical achievement, offering new insights into the field of online education. Understanding how self-regulated learning, mathematical mindset, and academic achievement interact is essential for improving students’ academic success, particularly in the underexplored context of Chinese higher education.

## Literature review

### Mathematics learning among college freshmen

Mathematics learning among college freshmen presents unique challenges and opportunities, as these students transition from high school to a more demanding academic environment. First-time freshmen, typically recent high school graduates, must navigate the complexities of college-level mathematics, which is often more rigorous than their high school coursework (Goodman et al., 2011). This transition is compounded by the pressures of academic achievement, social adjustment, and emotional stress (Deberard et al., 2004; D’Lima et al., 2014). One significant problem faced by freshmen is the gap in their mathematics readiness, highlighted by disparities between high school transcripts and the requirements of college mathematics courses (Hegedus et al., 2016). This puts students who have not taken advanced mathematics courses in high school at risk of struggling with college-level demands (Champion and Mesa, 2018; Froiland and Davison, 2016).

In Chinese universities, freshmen experience significant stress due to high familial and societal expectations, negatively impacting their mental health and academic success (Lei et al., 2022; Tang et al., 2023). Incorporating positive education strategies can alleviate this stress by emphasizing psychological well-being, engagement, and meaningful learning experiences. By promoting a supportive learning environment, students’ motivation and resilience can be enhanced, improving their persistence in challenging subjects like mathematics. Longitudinal studies have indicated that freshmen often experience notable changes in their mathematics performance over the course of their first year in college, with many showing a decline initially due to increased rigor, followed by gradual improvement as they adapt to new learning environments and strategies (Krumrei-Mancuso et al., 2013; Sax et al., 2002). Understanding these performance changes is crucial for identifying periods of vulnerability where students are most likely to struggle and implementing timely interventions (Zimmerman, 2002). Additionally, engagement and motivation might be further lowered if students fail to see real-world applications of algebra and calculus, leading to poor performance (Hiebert and Lefevre, 2013; Huang et al., 2017). This issue is compounded by inadequate study habits, especially in self-regulated learning (SRL), planning, and management skills, and reluctance to seek help, which significantly hinders their learning achievement (Kitsantas et al., 2021; Ye et al., 2022; Tang et al., 2023). Research also highlights that students who effectively implement self-regulated learning strategies tend to demonstrate significant positive changes in their mathematics performance over time, particularly in areas such as problem-solving accuracy and conceptual understanding (Pintrich and De Groot, 1990).These compounded difficulties underscore the multifaceted nature of the challenges that freshmen face in mathematics learning, highlighting the need for targeted support and interventions to help them succeed.

### Application of mindset theory in college mathematics education

Studies have consistently demonstrated that adopting a growth mindset—the belief that intelligence and abilities can be developed through effort, effective strategies, and support—significantly enhances students’ resilience and engagement, thereby improving their academic achievement in mathematics (Dweck, 2006, 2017; Duckworth et al., 2007). For example, Boaler (2013) found that students encouraged to adopt a growth mindset exhibited significantly higher levels of motivation and engagement, which translated into better academic outcomes. Similarly, Good et al. (2003) demonstrated that short-term interventions promoting a growth mindset could substantially improve students’ mathematics test scores, particularly among those who struggled initially. Building on these findings, it is important to distinguish between different types of mindsets that can influence mathematical learning. A fixed mindset refers to the belief that abilities are static and cannot be changed, whereas a growth mindset involves the belief that abilities can be developed through effort, effective strategies, and learning opportunities (Dweck, 2006). Mathematical mindset, as proposed by Boaler (2016), builds upon the growth mindset specifically in the context of mathematics. It emphasizes resilience, flexibility, and the value of learning from mistakes as essential aspects of mathematical learning. Unlike a general growth mindset, which applies across various domains, a mathematical mindset is tailored to foster positive attitudes towards mathematics, encouraging persistence through challenges.

Further studies have corroborated these findings, reinforcing the connection between growth mindset interventions and improved academic achievement in mathematics. Yeager et al. (2019) conducted a large-scale experiment demonstrating that mindset interventions not only enhance students’ mathematics grades but also bolster their resilience and capacity to cope with challenges. Similarly, Schmidt et al. (2015) found that feedback emphasizing effort and strategy rather than inherent ability significantly boosts students’ motivation and achievement in mathematics. Blackwell et al. (2007) demonstrated that educating students about the growth potential of intelligence leads to substantial improvements in mathematics course achievement. The broad applicability of mindset theory was further supported by Rattan et al. (2012), who found that growth mindset education increases students’ persistence through mathematical challenges, leading to better long-term achievement. Additionally, Esparza Masana and Cebrián (2024) showed that incorporating real-world applications and problem-solving tasks in mathematics courses reinforces a growth mindset, resulting in greater student engagement and effort. Despite these positive outcomes, there is a lack of research on how online interventions can effectively promote a mathematical mindset among college freshmen, especially within the context of Chinese higher education. Understanding how to leverage technology to foster mathematical mindsets could provide valuable insights for improving mathematics learning outcomes.

### SRL among college freshmen in mathematics

Empirical studies have highlighted the substantial impact of SRL strategies on freshmen’s achievement in mathematics. Sun et al. (2018) found that freshmen trained in SRL strategies outperformed their peers in introductory mathematics courses. Nota et al. (2004) also observed that freshmen who employed SRL strategies, such as goal-setting and self-monitoring, achieved higher academic success in mathematics. Additionally, Kramarski and Mizrachi (2006) revealed that freshmen who used metacognitive SRL strategies, including self-questioning and self-explanation, demonstrated better problem-solving skills and higher mathematics achievement. These findings align with those obtained by broader research indicating a strong positive correlation between SRL strategies and academic achievement in mathematics (Dignath et al., 2008; Zimmerman, 2002). SRL strategies enhance students’ problem-solving skills, adaptability, and persistence through challenging tasks (Cleary and Chen, 2009; Schunk, 2005). Self-monitoring and self-evaluation enable students to identify effective strategies and areas for improvement, fostering a deeper understanding of mathematical concepts (Boekaerts and Corno, 2005; Pintrich, 2004). However, there is a notable gap in research concerning how the form of online intervention can be used to effectively enhance SRL among college freshmen. Most existing studies have focused on in-person learning environments, leaving the unique dynamics and challenges of delivering SRL interventions through online formats largely unexplored (Hattie and Donoghue, 2016). Additionally, online platforms may present distinct challenges that require tailored SRL strategies to support student engagement and motivation (Artino, 2007; Broadbent and Poon, 2015). Online learning environments, especially those without a structured schedule or direct supervision, can make it difficult for students to self-regulate effectively, which is particularly true in complex subjects like mathematics (Huang et al., 2017). Further investigation is needed to understand how online formats, as a form of delivering SRL interventions, can effectively promote SRL behaviors and support student achievement in mathematics. This becomes especially critical given that students often struggle with self-regulation in less structured environments such as online learning. The National Council of Teachers of Mathematics (NCTM) underscored the importance of SRL by advocating for goal-setting, reflective thinking, active engagement in problem-solving, and perseverance (National Council of Teachers of Mathematics, 2014). Practical strategies to enhance SRL in mathematics include creating study schedules, seeking additional resources, developing focus techniques, establishing supportive learning environments, and regularly reviewing problem sets and exams (Zimmerman and Martinez-Pons, 1990). These SRL skills are teachable and lead to significant improvements in mathematical achievement and resilience (Cleary and Zimmerman, 2004; Perels et al., 2009). By utilizing online platforms as a medium to deliver SRL strategies, educators can better equip college freshmen with the critical skills necessary for success in mathematics and beyond, particularly in an increasingly digital educational landscape. Despite numerous studies emphasizing the importance of supporting freshmen’s transition to university-level mathematics learning and the demonstrated effectiveness of mathematical mindset interventions, significant research gaps remain. Mathematical mindset plays a crucial role in fostering SRL by shaping students’ attitudes toward challenges and their belief in their ability to improve through effort (Dweck, 2006; Zimmerman and Schunk, 2011). In particular, there is limited understanding of how these interventions can be effectively implemented within online formats for Chinese university students, and how this mode of delivery influences SRL and mathematical achievement in such contexts. Moreover, by encouraging a growth-oriented perspective, mathematical mindset interventions can enhance students’ willingness to engage in SRL behaviors, which has shown promise in enhancing engagement and motivation. However, their long-term effects on academic success and how they can be effectively integrated into existing higher education systems remain underexplored.

### Current study

Building upon the gaps identified in the literature, this study examines the impact of an online calculus tutorial designed to foster both a mathematical mindset and self-regulated learning (SRL) among freshmen in Chinese higher education. By integrating Dweck’s (2006) mindset theory with SRL strategies in an online learning environment, this research seeks to address the paucity of studies exploring these constructs collectively. Specifically, below research questions are addressed in this study:

*RQ1*: How do mathematical mindset interventions influence the relationships between freshmen’s self-regulated learning behaviors and mathematical performance in Chinese higher education?

*RQ2*: What is the effect of mathematical mindset interventions on freshmen’s overall academic achievement in mathematics, including the development of self-regulated learning behaviors?

**Figure 1:**
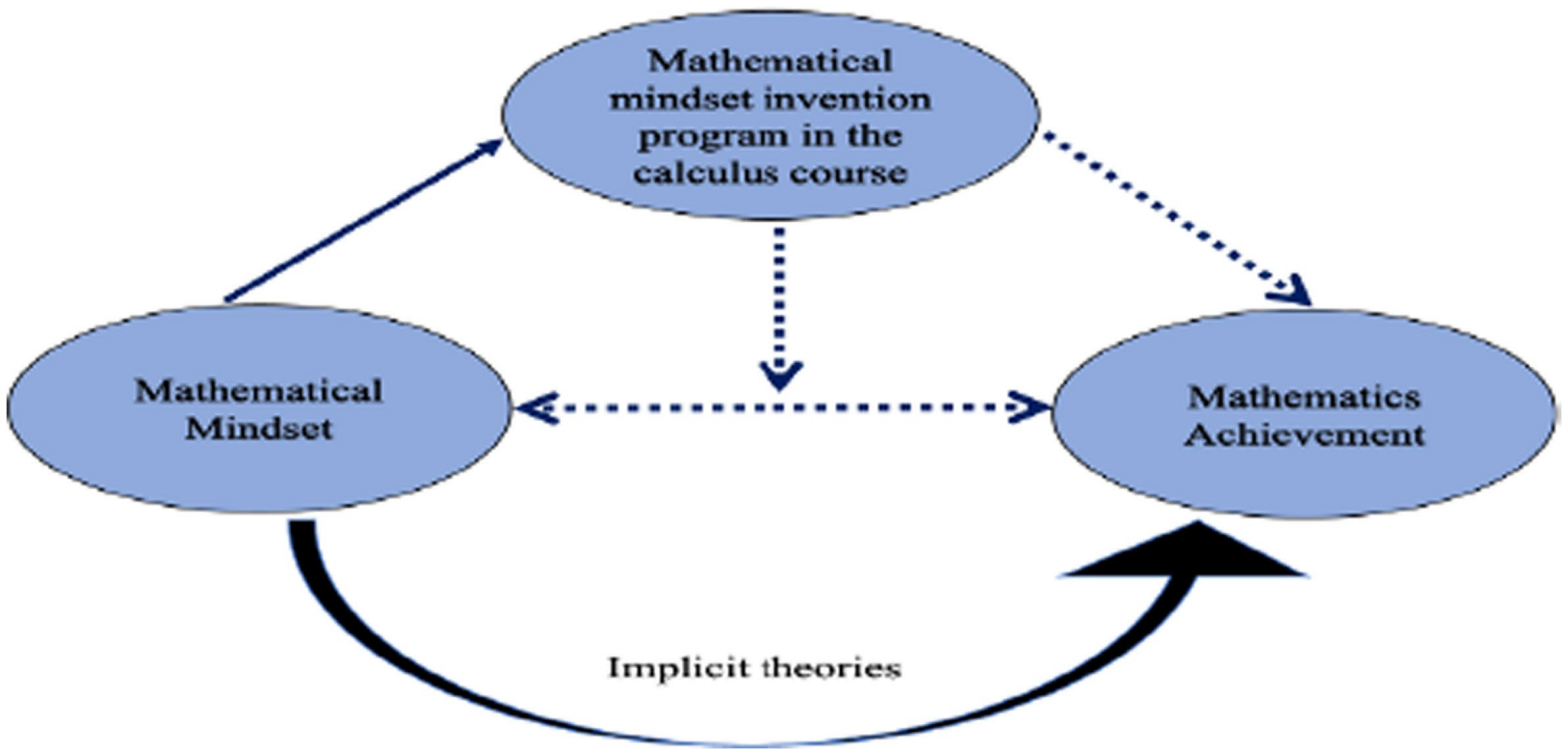
The conceptual model of the present study.

The reviewed literature informs this study by providing context, shaping the research question, and suggesting data collection techniques. Mathematical mindset interventions have recently gained attention for their potential to improve students’ attitudes, motivation, and achievement in mathematics. Studies, including those by Dweck et al. (2014), have shown that interventions emphasizing the malleability of intelligence and the importance of effort and persistence significantly enhance students’ motivation and academic performance (Dweck and Leggett, 1988). Approaches like educating students about neuroplasticity, using the term “yet” to signal ongoing learning, and highlighting mistakes as part of the learning process have proven effective (Opfer and Pedder, 2011). For Chinese freshmen, calculus is particularly crucial, yet many rely heavily on rote memorization and procedural knowledge, which hinders their problem-solving abilities (Fan and Zhu, 2004). Figure 1 illustrates the conceptual framework, which focuses on the relationship between mathematical mindset, the intervention experiment, and mathematics achievement. Established research suggests that mindset can be influenced by interventions, as indicated by the solid line, but the outcomes of this specific intervention and its effect on SRL and achievement remain unknown, represented by the dotted lines. We hypothesize that students who participate in this intervention will exhibit significant improvements in their SRL behaviors, such as goal setting and resilience, and achieve higher academic performance in calculus compared to those who do not receive the intervention. This study seeks to investigate these relationships and understand how fostering a mathematical mindset through targeted interventions can influence SRL and enhance mathematics performance.

## Method

### Participants

**Figure 2:**
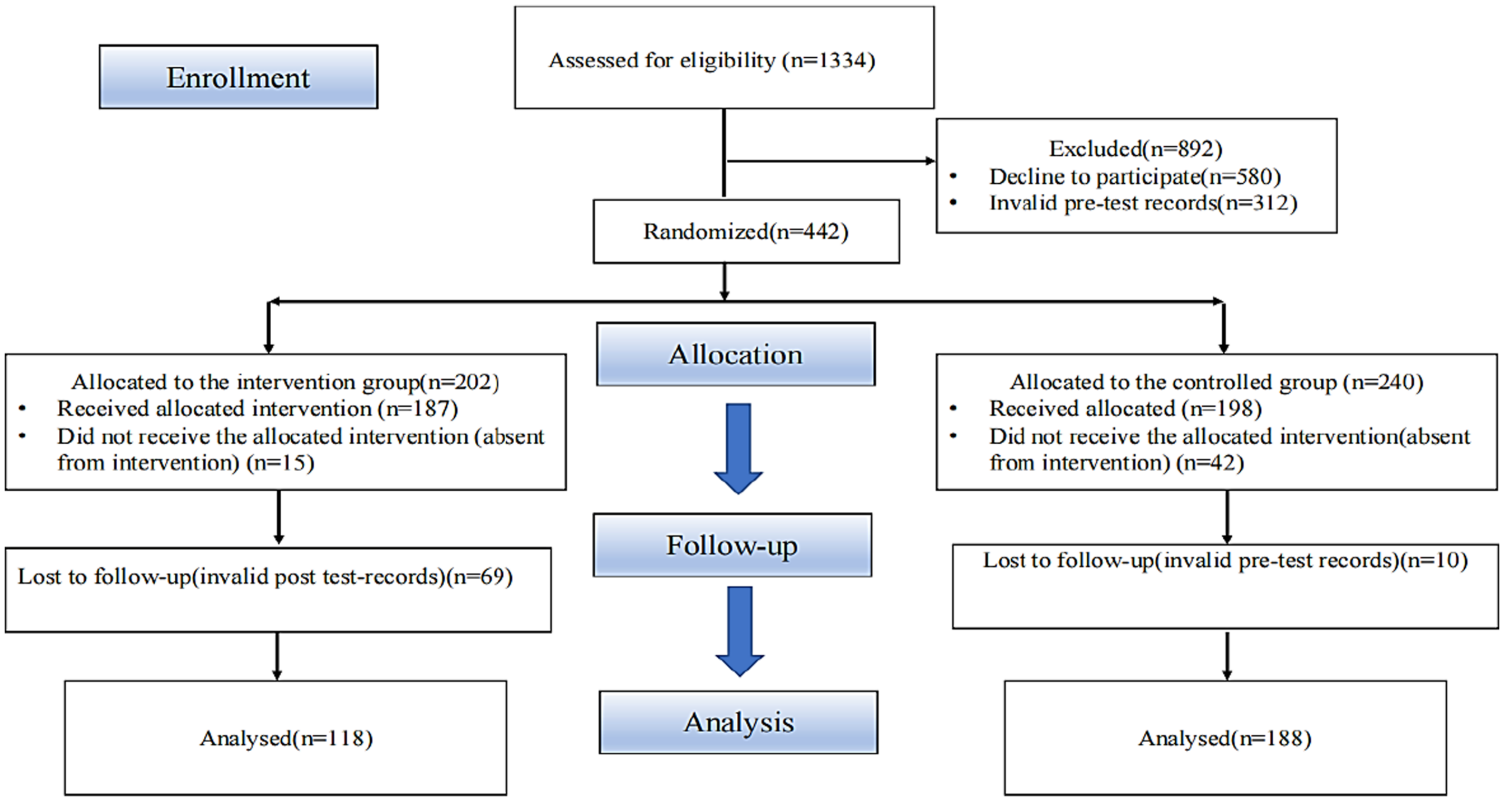
Flowchart of participation.

This study was conducted at a university in Guangdong, China. An initial pool of 1,334 students was considered eligible for the study. However, 892 students were excluded for various reasons: 580 declined to participate, and 312 had substantial issues with pre-test data. For example, some students provided incomplete responses, while others gave contradictory answers, such as selecting both “Strongly Agree” and “Strongly Disagree” for the same question. Thus, only 442 students were eligible to participate. To minimize potential biases and ensure that each group represented the overall student population, we used R software to allocate participants randomly to two groups: intervention and control. The randomization process considered varied student information, such as age, gender, and academic background, to ensure that both groups were comparable. Through this process, 202 participants were randomly assigned to the intervention group and 240 to the control group. However, due to withdrawals and data completion issues, the final groups comprised 118 participants in the intervention group (who received the intervention) and 188 in the control group who did not receive the intervention (see Figure 2). All participants were first-year students, distributed across four distinct majors: Business, Arts, Technology, and Finance. In the intervention group, the gender distribution was balanced, with 15 female students in each major (total intervention female = 60). Among male students, 14 students enrolled in each of the Arts and Finance majors, and 15 students enrolled in each of the Business and Technology majors (total intervention male = 58). The control group had a nearly equal gender distribution, with 23 male students (total control male = 92) and 24 female students (total control female = 96) in each major.

## Materials

### Intervention

**Figure 3:**
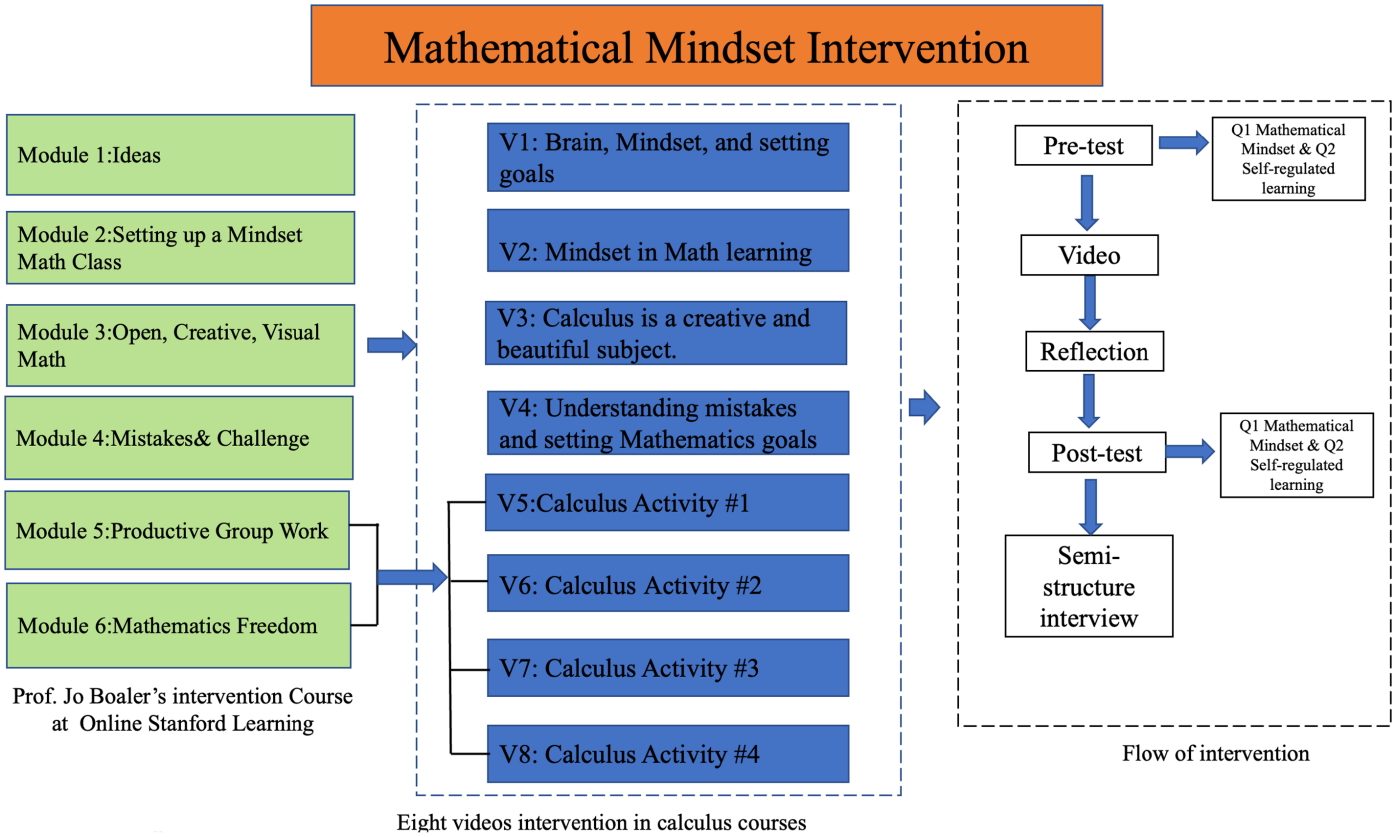
Mathematical mindset intervention.

In January 2023, 202 first-year students in the intervention group participated in a two-week online video series based on the mathematical mindset intervention module, which was extracted from Jo Boaler’s online course at Stanford Online Learning (Boaler, 2023). This intervention is directly grounded in Boaler’s mathematical mindset theory, which emphasizes that mathematical abilities are not fixed but can be developed through effort, persistence, and learning from mistakes. By incorporating these principles, the intervention specifically aimed to help students who had completed their calculus coursework online during the 2022 pandemic to build a growth mindset and enhance their SRL skills. As shown in Figure 3, the mathematical mindset intervention included six key modules (green boxes) and eight videos (blue boxes). After watching each video, the students participated in reflective activities (white box on the right).

Video 1 (V1) and Video 2 (V2) introduced the students to a mathematical mindset by explaining the brain and mindset and their relevance to learning calculus, as well as the relationship between mindset and goal-setting within the context of mathematics education. These videos are part of Module 1, which lays the foundation of growth mindset theory and its application to mathematics learning, drawing directly on Boaler’s (2016) work on the importance of believing in one’s ability to grow through effort and persistence. Video 3 (V3) delved into the idea that calculus is not merely a technical subject but also a creative and beautiful discipline. This video is aligned with Module 2, which is dedicated to changing students’ perspectives on calculus by highlighting its creative aspects. Boaler’s emphasis on exploring multiple solution paths and viewing mathematics as an inherently creative field is central to this module, encouraging students to break away from rote memorization and instead appreciate the beauty of mathematical thinking. Video 4 (V4) highlighted the role of calculus in fostering creative problem-solving skills and its application across various domains. This video supports Module 3, which aims to connect mathematical concepts with real-world applications and to foster creative thinking. The content here extends Boaler’s idea of cultivating an environment where students see the practical applications of mathematics, thereby reducing the anxiety around abstract concepts. Video 5 (V5) focused on activities that encourage students to see mistakes as valuable learning opportunities. Through individual activity, students worked in groups to analyze errors, set personal goals, and learn to persist through challenges together. This aligns with Boaler’s advocacy for embracing struggle as a part of effective learning. Video 6 (V6) provided a comprehensive overview of the calculus courses. Using group activities, they explored different calculus concepts. This was designed to encourage students to solve problems collaboratively and share diverse approaches, which deepens their understanding of the subject (Leikin, 2009). Video 7 (V7) emphasized mathematical freedom, encouraged students to design their own calculus problem-solving methods, and fostered creativity and engagement (Silver, 1997). This is another reflection of Boaler’s (2016) idea that students should have the freedom to explore mathematics in their own way, thus enhancing engagement and deeper understanding. The series concluded with Video 8 (V8), which revisited mindset, goals, and self-regulated learning, integrating themes of group work and mathematical freedom with calculus knowledge, such as creating a calculus mind map. This video emphasizes group work, mathematical freedom, and creating a calculus mind map to visually connect learned concepts.

#### Pre-and post-surveys

To observe the intervention’s impact, pre-and post-tests were conducted during the midterms (pre) and finals (post) using the online survey platform Wenjuanxing. Both the pre- and post-questionnaires were divided into three key parts: mathematical mindset, self-regulated learning, and mathematics achievement.

#### Mathematical mindset questionnaire

The 15 mathematical mindset questions were adapted from Boaler’s mathematical mindset questionnaire (Boaler et al., 2018), which has been used in many professional training courses (Boaler, 2023). It is divided into two key dimensions: beliefs about the plasticity of intelligence and challenge coping. Participants responded to each item using a six-point Likert scale ranging from “Strongly Disagree” (1) to “Strongly Agree” (6). In both the pre-test and post-test questionnaires, four items were designed such that higher scores indicated a stronger mathematical mindset (Q9, Q11, Q15, and Q17), while eleven items were designed such that lower scores indicated a stronger mathematical mindset (Q8, Q10, Q12-Q14, Q16, Q18-Q22) items were reverse scored. The internal consistency reliability was confirmed with Cronbach’s alpha coefficients of 0.73 at the pre-test and 0.96 at the post-test.

#### Self-regulated learning

The self-regulated learning questionnaire contained 24 questions adapted from Broadbent et al.’s (2023) Self-regulated Learning-online (SRL-O) questionnaire. This instrument assessed students’ self-regulated learning strategies across six key dimensions relevant to online learning environments: effort regulation (items 1–4), academic self-efficacy (items 5–8), planning and time management (items 9–13), online task strategies (items 14–18), intrinsic motivation (items 19–23), and metacognition (items 24–28). Participants responded to each item using a seven-point Likert scale ranging from “Strongly Disagree” (1) to “Strongly Agree” (7), with higher scores indicating greater use of the respective self-regulated learning strategy. Subscale scores were calculated by averaging the responses within each dimension, and an overall SRL score was obtained by summing the subscale scores, with higher total scores reflecting greater self-regulation in learning. The internal consistency reliability of the questionnaire was assessed using Cronbach’s alpha, yielding an overall alpha of 0.98 at the pre-test and 0.977 at the post-test, indicating excellent reliability (George and Mallery, 2003).

#### Mathematics achievement

The mathematics achievement questionnaire comprised 10 multiple-choice calculus questions sourced from university exams over the past 5 years, where higher scores indicated higher mathematical achievement.

#### Interviews

To gain deeper insights into the intervention’s effects on learning trajectories and mathematical mindsets, 18 students from both groups were selected for semi-structured interviews. These interviews drew on seminal works by Yeager et al. (2019), Blackwell et al. (2007), and Hattie (2009) to validate the program’s impact. Students were selected through stratified random sampling and asked a set of six core questions regarding their experiences with SRL and mathematical mindsets, which are provided in Appendix. The interviews were conducted in a comfortable setting, ensuring participants felt at ease. To ensure credibility and clarity, the interview questions were reviewed by experts in the field, and the analysis involved two researchers seeking consensus on the findings.

### Procedure

Data collection involved surveys, mathematics assessments, and interviews. Students in both the intervention and control groups participated in the pre-and post-tests, which covered self-regulated learning, mathematical mindset, and calculus problems from past midterm exams. The students in both groups used the same questionnaires. Participants in the intervention group, who followed an online video learning intervention, were assessed for changes in mathematical mindset. Eighteen students (9 from each group) participated in the post-survey interviews.

### Data analysis

This study employed an explanatory sequential mixed-methods approach to investigate mathematical mindset and self-regulated learning among first-year undergraduates. Incomplete surveys were managed using list wise deletion, a widely accepted technique in survey-based research to maintain data integrity when dealing with missing responses (e.g., Allison, 2009; Little and Rubin, 2019). This step was performed before conducting any statistical analysis to ensure that subsequent analyses were based on complete datasets. Descriptive statistics, including means, standard deviations, and correlations, were calculated to provide an overview of the data. Linear regression to examine the relationships among key variables, such as self-regulated learning and mathematical mindset. T-tests and *p*-values quantified the significance of changes, while R-squared values from regression models measured the intervention’s impact. Structural equation modeling (SEM) analysis was also performed to elucidate the impact of SLR on mathematical mindset, as well as changes in these relationships before and after the intervention. Following the quantitative phase, qualitative data were gathered through semi-structured interviews with 18 students, selected based on academic achievement, to gain the relationship between a mathematical mindset and SRL.

Stratified sampling was used to account for gender balance and academic achievement levels—lower, medium, and higher—ensuring a representative sample. The semi-structured interviews offered deeper insights into students’ mathematical experiences, self-regulated learning strategies, and mindset development. The 18 students were chosen from a total population of 306 (188 control and 118 intervention), ensuring equal representation from both groups. This sample size was guided by the principle of saturation, where further interviews do not yield new insights, typically achieved within 12–20 interviews. Conducted via Tencent Meeting, the interviews lasted 20 min and were analyzed for emerging themes related to students’ learning strategies and mindset (Boeije and Willis, 2013). All interviews were in Chinese and translated into English with the assistance of bilingual experts to ensure accuracy and facilitate cross-cultural analysis. Using Srivastava and Thomson’s (2009) framework, qualitative data were systematically analyzed through familiarization with the data, coding, and identifying key themes. Themes were refined through an iterative process in which patterns were reviewed, and connections between themes were visualized. By integrating these quantitative and qualitative findings, the study provided a comprehensive understanding of how the intervention influenced students’ attitudes toward mathematics, supporting the development of a growth-oriented learning environment.

## Results

### Descriptive statistics and significance testing

For self-regulated learning, the control group showed no significant difference in mean scores (*t* = −3.04, *p* = 0.136) from the pre-test (*M* = 138.3, *SD* = 22.75) to the post-test (*M* = 144.4, *SD* = 21.17). In contrast, the intervention group exhibited a significant improvement in SRL (*t* = 2.13, *p* = 0.041), with mean scores increasing from pre- (*M* = 140, *SD* = 26.13) to post-intervention (*M* = 147, *SD* = 25.50). For mathematical achievement, the control group showed no significant increase in mean scores (*t* = −3.56, *p* = 0.090) from the pre-test (*M* = 11.35, *SD* = 5.66) to the post-test (*M* = 13.25, *SD* = 6.03). The intervention group showed a slight but significant improvement (*t* = 2.05, *p* = 0.047), with mean scores rising from pre- (*M* = 12.32, *SD* = 4.95) to post-intervention (*M* = 12.45, *SD* = 5.96). For mathematical mindset, the control group showed a significant increase in mean scores (*t* = 4.45, *p* < 0.001) from the pre-test (*M* = 36.85, SD = 8.07) to the post-test (*M* = 39.76, *SD* = 6.14). The intervention group showed a slight but significant improvement (*t* = 7.48, *p* < 0.0001), with mean scores rising from pre- (*M* = 36.37, SD = 6.00) to post-intervention (*M* = 39.76, *SD* = 2.35). For mathematical mindset (higher), the control group showed no significant change in mean scores (*t* = −0.72, *p* = 0.48) from the pre-test (*M* = 18.1, *SD* = 2.55) to the post-test (*M* = 17.94, *SD* = 2.31). The intervention group showed no change (*t* = 0.00, *p* = 1.00), with mean scores remaining the same from pre- (*M* = 18.1, *SD* = 2.36) to post-intervention (*M* = 18.1, *SD* = 2.55). These findings indicate that the intervention group showed significant improvement in both SRL and mathematical achievement among freshmen, although the improvement in mathematical achievement was slight.

### Relationships between relationships between key variables and regression analysis

Previous findings showed significant improvements in SRL and modest gains in mathematical achievement for the intervention group. To understand the factors behind these outcomes, this section explores the use of regression analysis, correlation matrices, and SEM to understand better the impact of mathematical mindset interventions on student outcomes. Regression analysis was specifically chosen because it helps determine the strength and direction of the relationship between self-regulated learning (SRL) and mathematical achievement, allowing us to identify whether improvements in SRL predict better academic performance. This approach provides a more quantitative perspective on how SRL contributes to outcomes, which is essential for testing the effectiveness of the intervention. These methods were chosen because they allow for a more nuanced examination of the complex relationships between variables and mediating effects, which might not be fully captured with traditional analysis methods. The regression analysis focused on post-test outcomes, particularly the influence of SRL (“poself”) on mathematics achievement (“poma”). Although the model showed a slight positive effect—where “poma” increased by 0.234543 for each unit increase in SRL post-test “poself”—the predictors were not statistically significant (*p*-values >0.05) and the model had minimal explanatory power (R-squared = 0.02435, adjusted R-squared = −0.006138), with an F-statistic p-value of 0.4976, underscoring the model’s lack of significance. These findings suggest that SRL alone does not significantly impact mathematics achievement, highlighting the need for further investigation into other influential factors.

**Figure 4:**
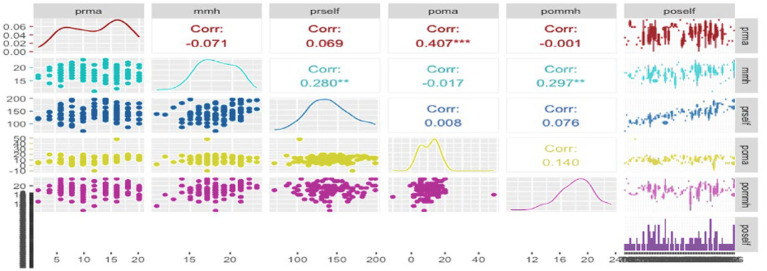
Correlation matrix of several significant relationships. The matrix displays correlations between several variables. prma, Pre-Test for Mathematical Mindset, mmh, Mathematical Mindset (where higher scores indicate a better mindset), mml, Mathematical Mindset (where lower scores indicate a better mindset), prself, Pre-Test Result for Self-Regulated Learning (SRL), poma, Post-Test for Mathematics Achievement, pommh, Post-Test for Mathematical Mindset, poself, Post-Test for Self-Regulated Learning (SRL). The colors in the matrix indicate different correlation directions and strengths: red represents negative correlations, while blue represents positive correlations. The density plots along the diagonal show the distribution of each variable. Asterisks denote levels of statistical significance: *p* < 0.05, *p* < 0.01, *p* < 0.001.

To complement the regression analysis, the correlation matrix provided a detailed examination of the relationships among the variables mathematics achievement “poma” and mathematical mindset variables—where higher scores on the questionnaire (“mmh”) indicated a better mindset and lower scores (“mml”) suggested a more favorable mindset—along with SRL post-test “poself.” Significant correlations, notably a strong positive correlation (Corr: 0.407), were observed between “poma” and “poself,” suggesting a consistent association between higher SLR and better mathematical achievement (see Figure 4). It shows a moderate positive correlation between pommh (mathematical mindset post-test) and poself (Corr: 0.297), suggesting that students with stronger self-regulated learning tend to have a more positive mathematical mindset. In contrast, the correlations between the mathematical mindset pre-test (prma) and both poself and pommh are weak, with values of 0.069 and − 0.001, respectively, indicating the limited influence of pre-test mindset on later outcomes. The density plots and scatter plots within the matrix further illustrate the data distribution and relationships between these variables, with the spread of poself reflecting variability in students’ self-regulation skills. This analysis underscores the critical role of self-regulated learning in improving both mathematical achievement and mindset, highlighting the importance of fostering SRL for better academic outcomes.

### Comparison of intervention and control group

**Figure 5:**
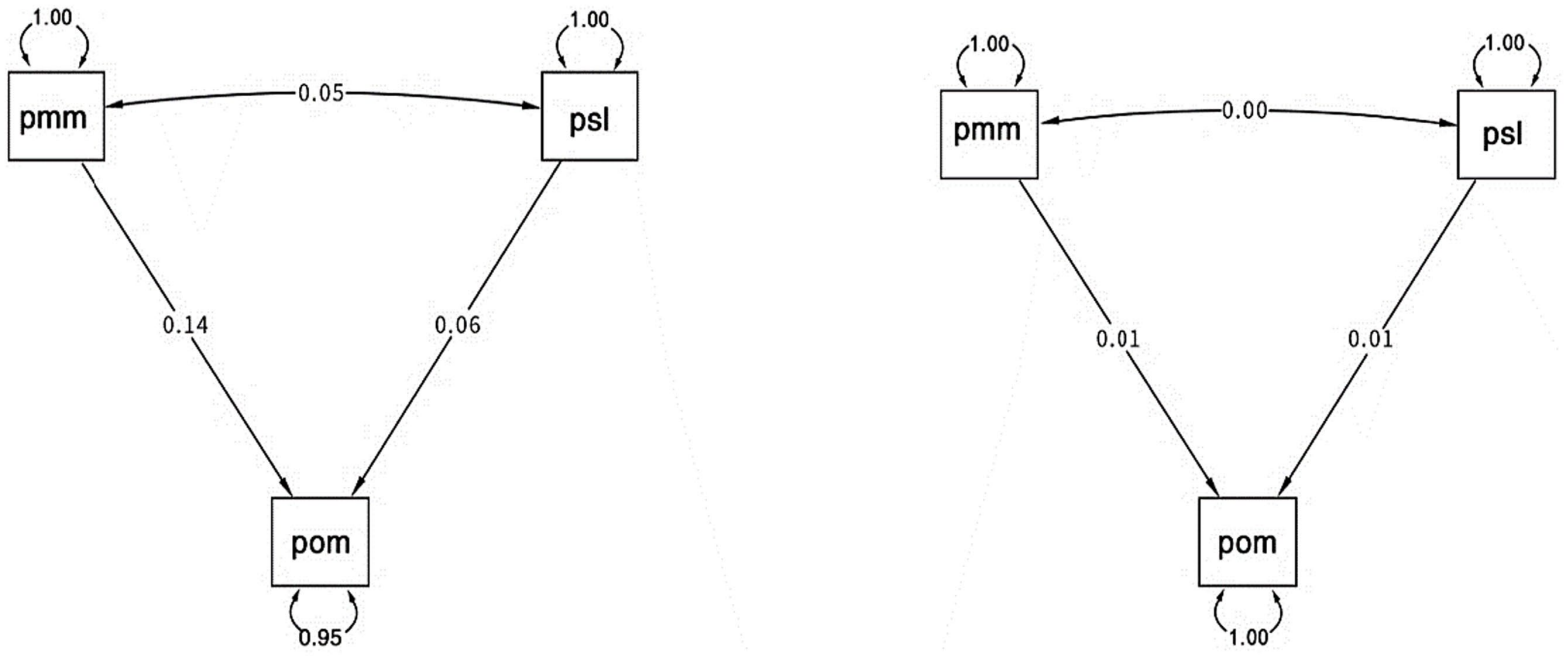
Comparison of structural equation models for intervention (left) and control (right) groups.

To evaluate the intervention’s effectiveness, we employed SEM to examine the relationships among mathematical mindset, self-regulated learning (SRL), and academic achievement. SEM analysis revealed that the intervention significantly enhanced participants’ mathematical mindset compared to the control group and that mastery of SRL, including online academic self-efficacy, positively predicted academic achievement. Additionally, SEM confirmed strong correlations among mathematical mindset, self-coordination, and academic achievement, highlighting the intervention’s role in strengthening these interconnected academic factors. The SEM path models for the intervention (left) and control (right) groups illustrate the relationships between mathematical mindset (pmm, abbreviated as such due to R’s display conventions), self-regulated learning (psl), and academic achievement (pom, also abbreviated by R) (see Figure 5).

The path coefficients in the intervention group suggest stronger relationships between variables, particularly between pmm and pom (0.14), compared to the control group where relationships are nearly negligible (0.01). The stronger path coefficients in the intervention group imply that the intervention had a positive effect on enhancing the relationships between mathematical mindset, self-regulated learning, and academic achievement. The SEM results, as shown in Figure 4, represent the relationships between academic achievement (pom), mathematical mindset (pmm), and self-regulated learning (psl). The analysis shows that academic achievement is positively associated with both mathematical mindset and self-regulated learning. However, only mathematical mindset (pmm) has a significant direct effect on academic achievement (estimate = 0.338, *p* < 0.05), whereas self-regulated learning (psl) does not (*p* = 0.133). The model demonstrates excellent fit, with CFI = 1.00, TLI = 1.00, RMSEA = 0.000, and SRMR = 0.000, clearly illustrating how a mathematical mindset plays a stronger role in academic achievement compared to self-regulated learning in this context. Since pmm and psl showed a closer relationship compared to their relationship with other variables, we further investigated the connection between individual items in mathematical mindset and self-regulated learning, as well as the correlation between the items themselves, to better understand how these two constructs related.

**Figure 6:**
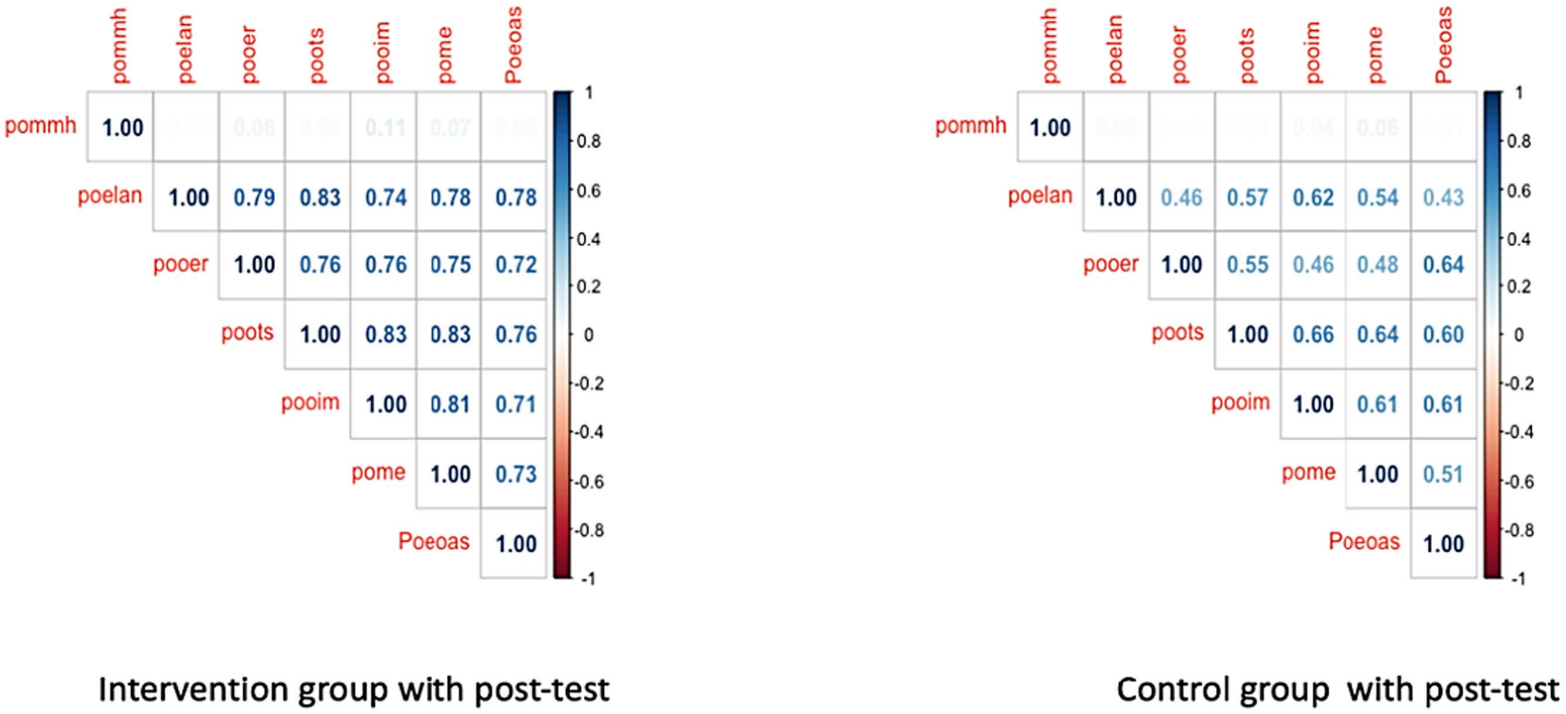
Correlation matrix comparison between intervention and control groups.

Figure 6 presents this comparison, highlighting the relationships between pommh (mathematical mindset) and key variables such as poelan (planning), pooer (effort regulation), poots (task strategies), pooim (intrinsic motivation), pome (metacognition), and Poeoas (academic self-efficacy). The intervention group exhibits significantly stronger correlations, with pommh correlating with poelan at 0.79 and poots at 0.83, and other variables such as proots, pome, and pooim reaching correlations as high as 0.83. This indicates that the intervention effectively strengthened the interconnections between mathematical mindset and self-regulated learning. In contrast, the control group shows weak correlations, underscoring the limited relationships between these factors without intervention. These findings demonstrate the intervention’s clear impact on enhancing the alignment between mindset and self-regulation, key elements for improving educational outcomes.

**Figure 7:**
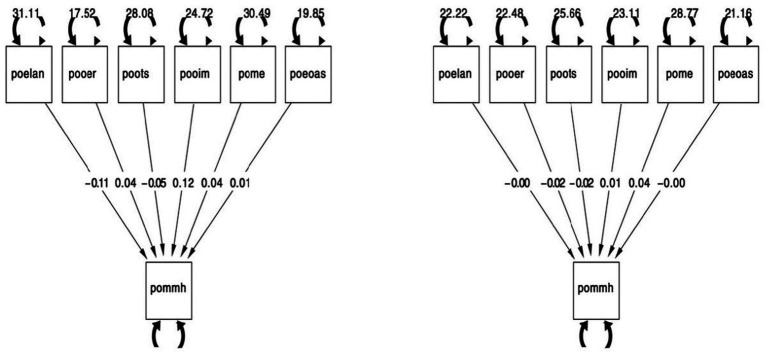
Mathematical mindset in the intervention (left) and control (right) group of the structural equation.

To further explore the dynamics between self-regulated learning components and mathematical mindset, we conducted an SEM analysis to examine how various predictors influence post-test mathematical mindset high (pommh), which reflects a strong mathematical mindset. As shown in Figure 7, the analysis investigates factors such as “post-test planning and management” (poelan), “post-test online effort regulation” (pooer), “post-test online task strategies” (poots), “post-test intrinsic motivation” (pooim), “post-test metacognition” (pome), and “post-test academic self-efficacy” (poeoas). The model demonstrates excellent fit with indices CFI = 1.00, TLI = 1.00, RMSEA = 0.000, and SRMR = 0.000, indicating no residuals. In the intervention group, pooim (intrinsic motivation) has the strongest positive effect on pommh (coefficient 0.12), highlighting its critical role in developing a strong mathematical mindset. Conversely, poelan (planning and management) shows a negative effect (−0.11), suggesting that rigid planning can hinder mindset growth. Other factors such as pome (metacognition) and poeoas (academic self-efficacy) also contribute positively, with coefficients of 0.04 and 0.01, respectively. In contrast, the control group shows weaker relationships, with coefficients close to zero across variables, indicating that the absence of intervention leads to minimal impact on mindset development. These findings underscore the intervention’s effectiveness in strengthening the connections between intrinsic motivation, self-regulated learning, and mathematical mindset, leading to significant improvements in students’ mindset and academic outcomes.

### Follow-up interviews

To thoroughly investigate the specific influence of mathematical mindset on student performance, we conducted follow-up interviews with 18 students from both the control and intervention groups (see Table 1). The purpose of including both groups in the interviews was to gain a comprehensive understanding of how different mindsets, including fixed mindset, growth mindset, and mathematical mindset are conceptualized by students and how these mindsets influence their learning behaviors and academic achievement. By comparing insights from both groups, we aimed to identify specific effects and differences that the intervention might have had and to explore whether and how these effects differ from the control group. This approach allows us to isolate the impact of the intervention more clearly by contrasting it with the baseline data from the control group, thereby providing a more nuanced analysis of the intervention’s effectiveness.

**Table 1:** Summary statistics for self-regulated learning, mathematical mindset, and achievement.

Categories	Pre		Post		*T*-test	Significance
	Mean (SD)	Range	Mean (SD)	Range		
Self-regulated learning	138.3 (22.75)	72–196	144.4 (21.17)	71–196	−3.04	*p* < 0.01
Intervention self-regulated learning	140(26.13)	71–196	147(25.50)	72–196	2.13	*p* < 0.05
Mathematical mindset(lower)	39.76(6.14)	21–60	36.85(8.07)	17–54	4.45	*p* < 0.001
Intervention Mathematical mindset(lower)	39.76(2.35)	17–60	36.37(6.00)	21–52	7.48	*p* < 0.001
Mathematical mindset (higher)	17.94(2.31)	10–23	18.1(2.55)	9–23	−0.72	n.s
Intervention Mathematical mindset (higher)	18.1(2.36)	11–23	18.1(2.55)	9–23	0.00	n.s
Mathematical achievement	11.35(5.66)	2–40	13.25(6.03)	0–40	−3.56	*p* < 0.01
Intervention Mathematical achievement	12.32(4.95)	2–20	12.45(5.96)	2–20	2.05	*p* < 0.05

*p* < 0.05, *p* < 0.01, *p < 0.001, n.s. (not significant).

Students were classified into three categories based on their mathematical performance changes: “no improvement” (score change from −10 to 0), “improvement” (score change from 1 to 8), and “high improvement” (score change from 9 to 16). Notably, a growth mindset does not guarantee improvement, as seen in Student 2, who demonstrated progress with a fixed mindset, whereas Student 3, despite having a growth mindset, did not improve. This highlights that other factors, such as teaching approaches, prior knowledge, or personal study strategies, can significantly influence academic performance.

Students with a fixed mindset who showed no improvement in academic achievement, such as S1_C_ and S7_I_, typically adhered to a passive learning approach. They faced significant difficulties in shifting to more active and SRL strategies, often relying heavily on external support and traditional methods. Conversely, students with a fixed mindset who demonstrated measurable improvement (improvement group), such as S2_I_, S11_C_, S13_C_, S14_I_, and S17_C_, began to incorporate elements of lifelong learning and problem-solving into their studies. This shift underscores the critical roles of self-motivation, resilience, and consistent practice in overcoming mathematical challenges. The following reflection by a student (S11C) illustrates how their approach to mathematical challenges evolved during the intervention, demonstrating the integration of self-regulated learning and strategic engagement.

“*When faced with mathematical challenges, I assess whether the problem aligns with my capabilities. If it does, I commit fully to solving it. If not, I focus on areas where I can be more effective. Attending lectures has been vital for active learning and developing personalized mnemonic strategies.*” This student’s reflection underscores the importance of aligning one’s capabilities with challenges and highlights how strategic engagement, such as attending lectures and using mnemonic strategies, contributed to their improvement.

Notably, S15_C_ and S16_C_ exhibited high improvement in fixed mindset through a robust determination to confront difficulties, actively seeking help, learning iteratively from mistakes, and engaging in continuous self-reflection. These cases highlight a significant transition toward a growth-oriented perspective within the fixed mindset category. Students moving toward a growth mindset, including S3_C_, S6_I_, S8_I_, S9_I_, and S12_C_, showed increased adaptability and proactive problem-solving skills. They evolved their learning approaches to become more dynamic, engaging actively in social learning and embracing educational challenges. This transition is also reflected in the following student’s (S16I) journey, which highlights the impact of supportive interventions and a shift in mindset on their approach to mathematics.

“*My journey in mathematics has seen significant growth. Initially, I struggled, but with supportive interventions and a shift in mindset, I developed a positive outlook. I now plan effectively and set precise goals. Tailored support and a changed perspective were pivotal in my success.*” This reflection illustrates how a positive shift in mindset, along with effective planning and tailored support, can significantly enhance a student’s engagement and success in mathematics.

This departure from conventional mathematical education was particularly evident in students like S10_I_, who made substantial progress by integrating self-regulation strategies, maintaining perseverance, and sustaining interest in mathematics despite encountering obstacles. These findings underscore the pivotal role of intrinsic motivation and the ability to adjust learning strategies to meet the demands of a changing educational environment and individual challenges.

Interestingly, we found that students who had a growth mindset also tended to have a mathematical mindset. The only exception was one student who had a growth mindset but did not exhibit a mathematical mindset. Only S18_I_ is not. Students with a mathematical mindset, such as S3_C_, S6_I_, S8_I_, S9_I_, and S12_C_, focused on enhancing their logical thinking and consistently applying learned patterns. This mindset facilitated a deeper understanding and more effective memorization of mathematical concepts. Students who demonstrated high improvement, such as S10_I_, highlighted the importance of self-regulation and persistence in adapting their learning strategies to their academic context. Those who exhibited high improvement, such as S4_C_, S5_I_, and S18_I_, excelled by aligning their learning strategies with curricular requirements and personal interests. Their improved mathematical performance was closely linked to their ability to maintain rigorous practice and continuously adapt their learning methodologies.

In addition to changes in mindset, substantial academic advancements were noted, particularly among S2_I_, S11_C_, S13_C_, S14_I_, and S17_C_. Their experiences, documented in Table 1, underscore the essential role of continuous learning and resilience in problem-solving. Adaptability and rigorous practice were key to overcoming mathematical challenges. Notably, S10_I_ exemplified the importance of self-regulation, perseverance, and sustained self-efficacy for mathematical progression. The epitome of elevated academic improvement was observed in students S15_C_ and S16_C_, who demonstrated determination, the propensity to seek assistance, learning from iterative mistakes, and the intrinsic preference for self-assessment were standout attributes. Concurrently, students S4_C_ and S5_I_ exhibited bespoke learning strategies tailored to curricular imperatives and individual proclivities. This dual alignment between SRL and a growth-oriented mindset underscores the mutable nature of mathematical competence, contingent on sustained practice.

To better understand these outcomes, it is essential to distinguish between fixed mindset, growth mindset, and mathematical mindset. A fixed mindset is characterized by the belief that abilities are static and unchangeable, as seen in students like S1c and S7I, who demonstrated no improvement and adhered to passive learning strategies. Conversely, a growth mindset involves the belief that abilities can be developed through effort, as observed in students like S10I, who improved by adapting their learning strategies and showing resilience. The concept of a mathematical mindset, specifically within the context of mathematics, goes beyond the growth mindset by encouraging creativity, resilience, and the value of learning from mistakes, which was evident in students like S4c and S5I, who showed high improvement by integrating rigorous practice and adaptability.

### Understanding mathematical mindset and its impact

**Figure 8:**
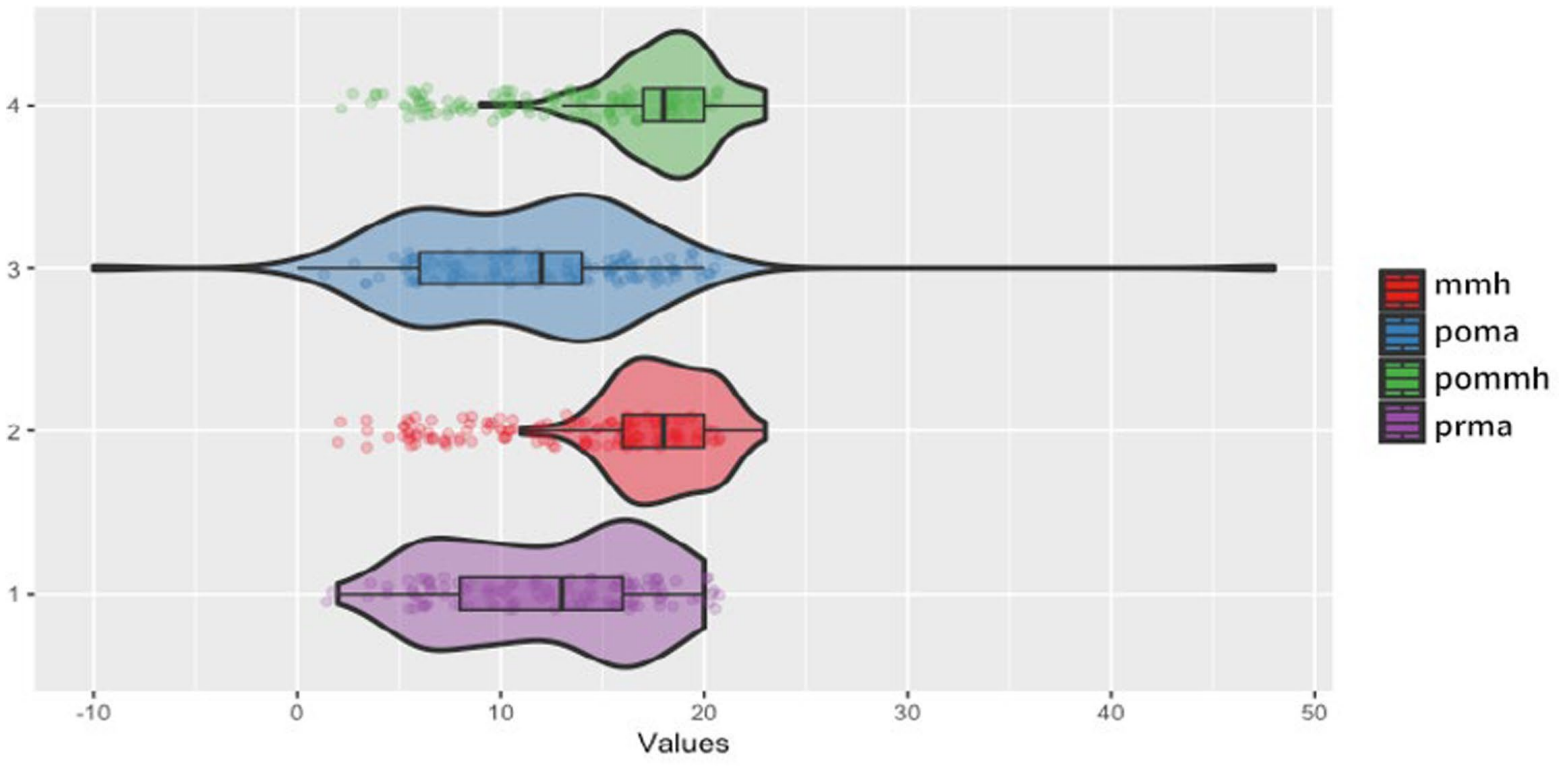
Violin plot of post-intervention performance and mindset for intervention group.

To understand mathematical mindset, we first compare changes in mathematical mindset and shifts in students’ thoughts (see Figure 8). We then investigate students’ perceptions of growth mindset, fixed mindset, and mathematical mindset, focusing on the distinctions and commonalities between these concepts to clarify how they affect learning and academic performance. Students were categorized into three groups based on their performance trajectories—those with significant improvement, stable performance, and significant decline. Additionally, they were divided into control (C) and intervention (I) groups to assess the specific impacts of the educational strategies, as detailed in Table 1. This comprehensive approach allows us to evaluate how different mindsets and performance trajectories interact with the implemented strategies.

Following this, we visualized the intervention group’s impact using the violin plot shown in Figure 8. This plot shows the distribution and spread of four critical variables: higher mathematical mindset (mmh, red), post-intervention mathematical achievement (poma, blue), post-intervention higher mathematical mindset (pommh, green), and pre-test mathematical achievement (prma, purple). Poma shows a broad spread, indicating diverse post-intervention achievement scores, while pommh has a high median and moderate spread, suggesting positive impacts on students’ mindsets. In contrast, mmh and prma have tighter clusters and lower medians, indicating more consistent and less varied scores. This visualization highlights how the intervention enhanced the mathematical mindset and diversified student achievement outcomes. Additionally, the plot identifies outliers, especially in mmh, with a few students scoring significantly higher or lower than their peers. Comparing the variables, poma and pommh exhibit broader distributions, reflecting greater variability in post-intervention outcomes and mindset improvements, contrasting with the tighter spread of prma, which indicates more uniform pre-test achievement scores. These findings suggest that the intervention led to varied responses in post-intervention mathematical achievement and mindset.

## Conclusion and discussion

### SRL as a driver of mathematical mindsets and academic achievement

Our study emphasizes the crucial role of SRL strategies in enhancing mathematical achievement and fostering a positive mathematical mindset among college freshmen. Our findings show that students who adopt logical thinking and systematically apply learned patterns experience significant improvements in their mathematical mindset (see Figure 8). The correlation analysis in Figure 3 further highlights strong positive relationships among SRL, mathematical mindset, and mathematics achievement, demonstrating how these elements collectively contribute to academic success. Additionally, Figures 5, 6 reveal that the intervention group’s mathematical mindset and SRL behaviors improved notably, with SRL showing significant gains and mathematical achievement showing slight improvement.

The intervention also fostered measurable growth in both mathematical thinking and SRL, with intrinsic motivation emerging as a particularly influential factor. Post-test data indicate that online intrinsic motivation had the strongest positive effect on the mathematical mindset, suggesting that students who are internally motivated to learn develop a more resilient and adaptable mathematical mindset. This finding aligns with Deci and Ryan’s (2000) definition of intrinsic motivation, which enhances students’ engagement with mathematical content, goal-setting, and perseverance. Students who are intrinsically motivated maintain focus and effort despite difficulties, fostering a resilient approach to learning mathematics.

Conversely, an overemphasis on rigid planning (“poelan”) was associated with a negative effect on mindset, suggesting that inflexible planning can hinder the development of a growth-oriented mindset. This insight underscores the need for flexibility in SRL strategies to support students’ ability to adapt to challenges in mathematics. Our interview data (see Table 1) provide further insight into the relationship between a positive mathematical mindset and strong SRL skills. Students with constructive attitudes toward mathematics and robust SRL skills approach mathematical challenges with confidence and adaptability, reinforcing the importance of these attributes in overcoming academic obstacles. Improvements in SRL—particularly in intrinsic motivation, effort regulation, and metacognition—corroborate Zimmerman’s (2002) framework, highlighting their role in academic success.

These insights demonstrate the transformative potential of targeted educational interventions in fostering a dynamic mathematical mindset and creating a culture of academic resilience. Our study shows that SRL strategies and a positive mathematical mindset substantially enhance students’ mathematical achievement. Educators should, therefore, prioritize these factors, implementing strategies that cultivate intrinsic motivation and self-regulation to empower students in mathematics and beyond.

### Enhancing and refining mathematical mindsets through strategic interventions

The results of this study highlight positive shifts in students’ mathematical mindsets following targeted interventions, addressing RQ1 by exploring how these interventions influence SRL behaviors and mathematical performance. Analysis of data presented in Table 2 and Figure 8, along with interview results, shows a notable shift in students’ mindsets, with many becoming more resilient and adaptable in overcoming mathematical challenges. Table 1 further illustrates that, when reflecting on their learning, students frequently used positive language, especially about their growing ability to tackle complex problems. This indicates a gradual shift toward a more growth-oriented mathematical mindset.

**Table 2:** Student traits and improvements in mindsets.

	Fixed mindset	
	Students	Characteristics from student interviews
No improvement	S1_c_, S7_I_	Emphasizing the shift from passive to active learning, their strategies for overcoming challenges, the role of support, and the importance of SRL.
Improvement	S2_I_, S11_c_, S13_c_, S14_I_, S17_c_	Emphasizing the philosophy of lifelong learning and problem-solving through a growth mindset, as well as the importance of self-motivation, resilience, flexibility, and consistent practice in overcoming challenges, particularly within mathematics learning.
High improvement	S15c, S16c	Emphasizing a determination to overcome challenges, the willingness to seek help and learn from mistakes, consistent practice of self-reflection and reassessment of one’s own understanding, and the resilience to keep trying despite difficulties.

	Students	Common traits
Growth mindset		
No improvement	S3_c_, S6_I_, S8_I_, S9_I_, S12_c_	Emphasizing learning in other areas is characterized by a more flexible approach, adapting to the nature of the field, problem-solving, and adopting social learning from peers.
Improvement	S10_I_	Emphasizing SRL is about adjusting and changing your learning strategies based on the environment and university context. When faced with difficulty or frustration, persistence and maintaining interest in the subject, alongside investing substantial time, are crucial. Continuous self-assurance of your capabilities is part of this self-regulatory process.
High improvement	S4_c_, S5_I_, S18_I_	Emphasizing adapting your learning strategies based on class requirements and your own interests, showing self-regulation in learning.
Mathematical mindset		
No improvement	S3_c_, S6_I_, S8_I_, S9_I_, S12_c_	Emphasizing on pattern recognition and logical thinking, mathematical mindset is about consistently understanding, memorizing, and applying solution templates while actively seeking help when needed.
Improvement	S10_I_	Emphasizing on growth and evolution through practice and training, challenging the fixed mindset for mathematics learning.
High improvement	S4_c_, S5_I_	Emphasizing on the belief that mathematics abilities can improve through practice.

c, control and I, intervention.

Inspired by Boaler et al. (2022), our intervention demonstrated a measurable improvement in students’ views of their abilities as malleable and capable of growth, fostering greater engagement and perseverance. These findings align with Linnenbrink and Pintrich (2003), who observed that growth-mindset interventions enhance motivation and resilience in structured academic settings, supporting RQ2 by showing how mindset interventions impact both academic achievement and the development of SRL behaviors.

Similarly, Smith and Jones (2010) reported that students focusing on procedural fluency still achieved high performance, especially in assessments emphasizing accuracy and memorization. This consistency underscores the value of understanding how different educational strategies can support students across various mindsets, as addressed in both RQ1 and RQ2. A particularly compelling finding is the substantial improvement in mathematical achievement among students who transitioned from a fixed to a growth mindset. Initially, many students perceived their mathematical abilities as fixed, leading to disengagement and reluctance to tackle challenges. However, the intervention encouraged these students to adopt a growth mindset, promoting the belief that effort and learning could improve their abilities. This shift led to higher grades and greater enthusiasm in tackling complex problems, aligning with RQ2’s focus on how mindset interventions affect academic achievement and SRL development.

Interestingly, some students with a fixed mindset also demonstrated high mathematical performance, likely due to reliance on procedural learning strategies such as repetition and memorization. These strategies can be particularly effective in traditional assessments focused on procedural accuracy (Schmidt and Vandewalle, 2012). This aligns with RQ1 by suggesting that while a growth mindset may foster a more adaptable understanding, a fixed mindset can still yield high performance under specific conditions depending on SRL behaviors and assessment structure. Furthermore, external factors like parental pressure and a structured learning environment may contribute to high performance despite a fixed mindset, creating conditions where students are motivated to achieve even if they do not believe in their growth capacity. These findings suggest that while a growth mindset generally supports deeper understanding and engagement, a fixed mindset can effectively achieve high scores in certain contexts. Understanding the balance between procedural mastery and conceptual understanding is crucial for designing interventions that support all students, as both RQ1 and RQ2 imply. Despite these positive outcomes, final interviews revealed that students’ understanding of a mathematical mindset remained somewhat superficial. To fully appreciate the impact of the intervention, it is essential to distinguish between a fixed mindset, a growth mindset, and a mathematical mindset (Dweck, 2006).

A fixed mindset is characterized by the belief that abilities are static and unchangeable, often leading to challenge avoidance and fear of failure. In contrast, a growth mindset reflects the belief that abilities can improve with effort and perseverance. The concept of a mathematical mindset, introduced by Boaler (2016), builds specifically on the growth mindset in the context of mathematics. It emphasizes resilience, adaptability, and the importance of learning from mistakes, encouraging flexibility and creativity in approaching mathematical problems. These distinctions also reveal potential cultural influences. For example, Chinese students often view mathematics as a series of logical procedures, shaped by educational practices emphasising rote learning and mastery of algorithms (Cai, 2003; Wong, 2017). This approach fosters procedural mastery but may limit flexibility and creativity. Our findings, shown in Table 1, indicate that while some students equate a mathematical mindset solely with logical reasoning, a broader mathematical mindset encompasses not only procedural fluency but also problem-solving, creativity, and adaptability. Cai et al. (2004) note that cultural expectations significantly shape students’ attitudes and beliefs toward mathematics, which impacts their approach to learning. In response, future interventions should redefine and expand students’ understanding of a mathematical mindset, as recommended by Boaler (2016), to include procedural skills alongside broader competencies.

By integrating these concepts into the curriculum, educators can help students develop a holistic understanding of mathematics, equipping them to approach challenges with both flexibility and innovation. This approach supports the findings in both RQ1 and RQ2, suggesting that a balanced focus on mindset and SRL strategies can foster academic resilience and a richer, more adaptable understanding of mathematics.

## Limitations and recommendations for practice

This study has several limitations that should guide future research and practice. One limitation is the absence of a follow-up period to assess the long-term effects of the two-month online video intervention. While the immediate impact on learning motivation was observed, the sustainability of these effects over time remains unknown. Future research should include follow-up assessments to determine whether changes in mindset and academic improvement persist. Additionally, the study’s relatively narrow sample—primarily focused on university students taking higher mathematics as a foundational course—limits the generalizability of findings. Expanding the sample to include students from various disciplines and institutions could broaden the applicability of the results.

Another limitation is the cross-sectional design, which captures only a snapshot of the intervention’s effects. Future studies could adopt a longitudinal approach with larger sample sizes to track students over time, providing deeper insights into how mindset interventions impact academic success and SRL behaviors across educational stages. Such an approach would allow for a more comprehensive exploration of the sustained influence of educational interventions on students’ mindset orientations and long-term academic outcomes. Mixed-method approaches are also recommended to gain richer, qualitative insights into the intricate dynamics of mathematical learning processes, ultimately contributing to a more nuanced understanding of effective educational practices. The role of teachers is crucial in implementing mindset-based educational interventions, though it is not a limitation *per se*. For teachers aiming to foster positive mathematical mindsets, comprehensive training is essential to ensure a solid understanding of mindset theory and SRL principles (Boaler, 2019). In addition, the intervention design should emphasize timely and personalized feedback, which is instrumental in reinforcing student engagement and confidence in challenging subjects like mathematics (Hattie and Timperley, 2007). Immediate feedback allows students to address errors early on, preventing misconceptions from solidifying, while personalized feedback caters to each student’s unique needs, making learning more targeted and effective.

To further support engagement and SRL development, incorporating animations and multimedia resources can make abstract mathematical concepts more accessible and interesting (Mayer, 2002). Encouraging students to develop SRL strategies helps them take control of their own learning, leading to improved outcomes (Zimmerman, 2002). Since many students find mathematics challenging, integrating real-life applications and problem-solving projects that connect mathematical concepts to practical scenarios can enhance relevance and reduce anxiety (Boaler, 2016). Embedding these strategies into the curriculum fosters a positive, resilient mathematical mindset, which is essential for sustained academic success.

## Declarations

In our study, we adhered to the highest ethical standards and obtained approval from the Human Research Ethics Committee (HREC) of The Education University of Hong Kong. Our research project, titled “Developing Students’ Mathematical Mindsets for Self-Regulated Learning: A Case Study of a Calculus Course in a Chinese University,” received approval under reference number 2022–2023-0193 for the period from January 5, 2023, to December 31, 2023. All participants provided informed consent, were informed of the research purpose, procedures, potential risks, and benefits, and were assured that their privacy and confidentiality would be maintained. (Ethical Approval No.: 2022–2023-0193).

## Data Availability

The original contributions presented in the study are included in the article/Supplementary material, further inquiries can be directed to the corresponding author.

## Appendix. Interview questions

1. Explain your concept of the growth mindset, fixed mindset, and mathematical mindset.
2. Explain your understanding of self-regulation. What are your self-regulated strategies, when you are learning mathematics? How to use it?
3. How would you describe your mindset when it comes to mathematics learning?
4. Where do you see you struggle in math, and what barriers prevent you from experiencing success?
5. Are you confident about learning math? Could you explain it?
6. What have you done to try and motivate yourself to learn mathematics?
